# The initial experience of electronic brachytherapy for the treatment of non-melanoma skin cancer

**DOI:** 10.1186/1748-717X-5-87

**Published:** 2010-09-28

**Authors:** Ajay Bhatnagar, Alphonse Loper

**Affiliations:** 1 Department of Radiation Oncology, University of Pittsburgh School of Medicine, Pittsburgh, PA USA; 2 Cancer Treatment Services - Arizona, 1876 East Sabin Drive, Suite 10, Casa Grande, AZ 85122 USA

## Abstract

**Background:**

Millions of people are diagnosed with non-melanoma skin cancers (NMSC) worldwide each year. While surgical approaches are the standard treatment, some patients are appropriate candidates for radiation therapy for NMSC. High dose rate (HDR) brachytherapy using surface applicators has shown efficacy in the treatment of NMSC and shortens the radiation treatment schedule by using a condensed hypofractionated approach. An electronic brachytherapy (EBT) system permits treatment of NMSC without the use of a radioactive isotope.

**Methods:**

Data were collected retrospectively from patients treated from July 2009 through March 2010. Pre-treatment biopsy was performed to confirm a malignant cutaneous diagnosis. A CT scan was performed to assess lesion depth for treatment planning, and an appropriate size of surface applicator was selected to provide an acceptable margin. An HDR EBT system delivered a dose of 40.0 Gy in eight fractions twice weekly with 48 hours between fractions, prescribed to a depth of 3-7 mm. Treatment feasibility, acute safety, efficacy outcomes, and cosmetic results were assessed.

**Results:**

Thirty-seven patients (mean age 72.5 years) with 44 cutaneous malignancies were treated. Of 44 lesions treated, 39 (89%) were T1, 1 (2%) Tis, 1 (2%) T2, and 3 (7%) lesions were recurrent. Lesion locations included the nose for 16 lesions (36.4%), ear 5 (11%), scalp 5 (11%), face 14 (32%), and an extremity for 4 (9%). Median follow-up was 4.1 months. No severe toxicities occurred. Cosmesis ratings were good to excellent for 100% of the lesions at follow-up.

**Conclusions:**

The early outcomes of EBT for the treatment of NMSC appear to show acceptable acute safety and favorable cosmetic outcomes. Using a hypofractionated approach, EBT provides a convenient treatment schedule.

## Background

The incidence of both non-melanoma and melanoma skin cancers has been increasing over the past decade. An estimated 2 to 3 million non-melanoma skin cancers (NMSC) occur in the U.S. each year,[1] which is greater than the estimated number of new cases of all other types of cancer combined[2]. If the rate of occurrence of NMSC per capita is similar in Europe, then approximately 4 million cases of NMSC could be expected in the European Union's population of 501 million people each year. In the U.K. alone, 84,500 cases of NMSC were registered in 2007, and this number was known to be an underestimate of the number of diagnosed cases[3]. According to the American Academy of Dermatology, 80% of NMSC lesions in the U.S. are basal cell carcinomas (BCC), and 16% are categorized as squamous cell carcinoma (SCC)[4].

A variety of modalities for the treatment of BCC and SCC are available, including surgery, radiation therapy and topical agents. Surgical options, including curettage with electrodessication, Mohs micrographic surgery, and surgical excision, are the most frequently used treatments, providing a high control rate and satisfactory cosmetic results[5-7]. However, some patients are not suitable candidates for surgery due to age or general health, and some cases of NMSC may not be optimally treated with surgery due to the potential for disfigurement. Aggressive cases of SCC may respond best to a combination of surgery and post-surgical adjuvant therapy[8]. Radiation therapy, including external beam and brachytherapy techniques, has been used as primary and post-surgical adjuvant therapy for NMSC. External beam radiation modalities have included superficial x-rays (45-100 kV), orthovoltage x-rays (100-250 kV), megavoltage photons, and electron beam radiation. Published studies report local control ranging from 87-100% at two to five years with excellent to good cosmetic outcomes reported in the absence of grade 4 toxicities[9-14]. Dose fractionation schemes for external beam radiation therapy are based on the size and location of the lesion and can take up to seven weeks of daily treatments for a 70 Gy prescription dose to be delivered in 35 fractions[9-13]. High Dose Rate (HDR) brachytherapy using skin surface applicators or surface molds can reduce the number of treatments and the duration of the treatment schedule. Kohler-Brock, et al., reported their 10-year experience with 520 patients with skin lesions mainly comprising SCC and BCC treated with standardized surface applicators and a remote afterloading HDR system. The dose per fraction ranged from 5-10 Gy delivered once to twice per week with a total dose ranging from 30-40 Gy. The recurrence rate was 8%, and there were no observed severe late radiation reactions[15]. Guix, et al., published their series of 136 patients with BCC or SCC of the face treated with surface molds and HDR brachytherapy using a radioisotope source (Ir-192) and showed a 5-year local control rate of 98% with no severe early or late complications detected[16].

**Figure 1:**
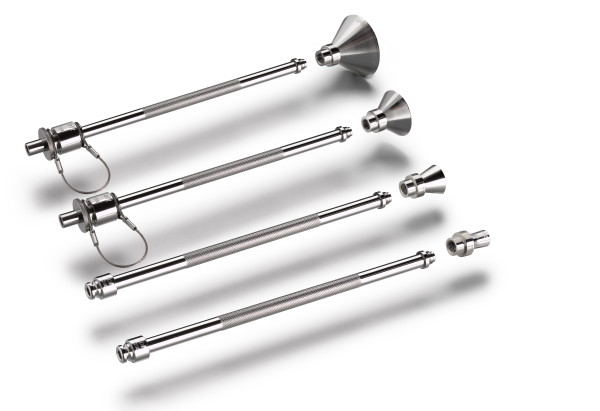
**EBT Surface Applicators for Use with the Axxent^® ^System**.

**Figure 2:**
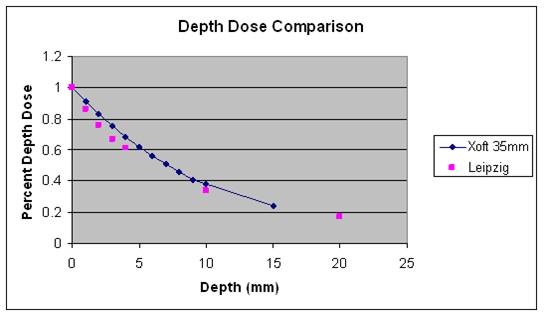
**Depth Dose Comparison of HDR EBT with HDR ^192^Iridium**.

**Figure 3:**
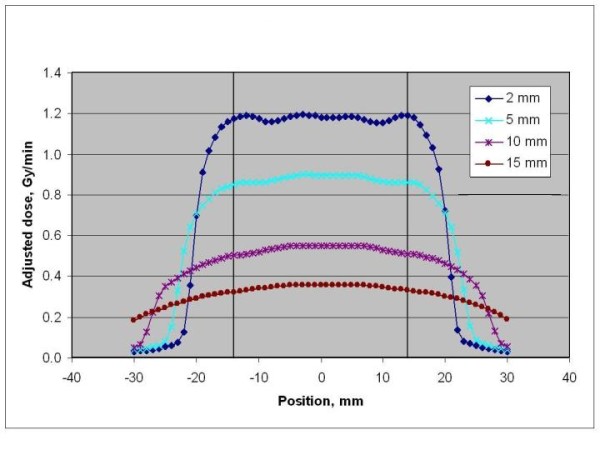
**EBT Source Beam Profile**.

Electronic brachytherapy (EBT) is the administration of HDR brachytherapy without the use of a radioactive isotope and with minimal shielding requirements due to the low energies utilized with this system. EBT treatments are delivered using the Axxent^® ^System controller, source and surface applicators (Xoft Inc., Sunnyvale, CA), which have been cleared by the United States Food and Drug Administration to deliver HDR X-ray radiation for brachytherapy. The EBT skin surface applicator weighs less than 2 pounds and appears similar to the Leipzig applicator used with HDR Iridium-192 (Ir-192) brachytherapy (Figure 1). Dosimetric analyses have been performed revealing similar depth dose profiles for these two surface applicators (Figure 2)[17-19]. However, data output for the beam profile measurements show superior beam flatness with reduced penumbra for the EBT surface applicator (Figure 3)[17-20].

The purpose of this manuscript is to report the initial experience, feasibility, and clinical outcomes of EBT using the Axxent System and surface applicators for the treatment of NMSC.

## Methods

All patients treated with EBT for NMSC at Cancer Treatment Services - Arizona from July 2009 through March 2010 were included in this study. This retrospective study was approved by Integriew Ethical Review Board. Data were collected retrospectively on a case report form from the medical records. Pre-treatment biopsy for NMSC had been performed on all patients to confirm the diagnosis prior to treatment. A series of digital photographs of the initial lesion on each patient was obtained.

### Simulation

Customized immobilization using a thermoplastic mask (Civco, Orange City, IA) for facial lesions and Vac-Lok™bags (Civco, Orange City, IA) for extremity lesions were used to immobilize and locate the area to be treated prior to administration of each fraction. The customized immobilization ensured constant and complete surface contact between the surface applicator and the skin lesion for the duration of the treatment. All patients also underwent CT scan of the treatment region to assess skin depth. A digital photo, illustrating the method of immobilization of the treatment area and a simulation of the set-up of the system prior to the first fraction, was taken.

A typical treatment area definition begins with an assessment of the visible surface lesion, known as the gross tumor volume (GTV). An additional margin to account for measurement uncertainty, profile edge effect, and uncertainty in applicator placement was added; this constitutes the planning target volume (PTV). An applicator diameter that was large enough to encompass the entire PTV was chosen.

### Treatment Planning

The objective of the treatment planning process for EBT using the surface applicators is to calculate a dwell time to deliver the prescribed dose at a specified depth. The process for EBT surface application treatments follows a similar approach as traditional Ir-192 HDR brachytherapy using the Leipzig applicators. In both modalities, a prescription depth and dose are chosen, an applicator size is selected, and the patient is treated for a dwell time. The key distinction between the two modalities is the calibration and calculations associated with the EBT 50 kV source versus the Ir-192 source.

After the applicator size is selected, a single dwell position is used to deliver the prescribed dose (D_prescribed_) to the prescription depth. The nominal dwell time (t_Nominal_), the calculated time to deliver the dose to the single dwell position, is calculated using the following factors: (Ď_nominal_) in Gy/min, based on the AAPM Task Group 61 report,[21] the percentage depth dose (PDD) and the prescribed dose (D_prescribed_). When following the TG-61 protocol, the source and surface applicator are calibrated as a set, and all measurements are dependent on the actual air kerma strength of the source used (AKS_Actual_) and the nominal air kerma strength (where AKS_Nominal _= 110,000 U). The actual dose rate (Ď_Actual_) must be converted to a nominal dose rate (Ď_Nominal_) as shown below.

$${Ď}_{\text{Nominal}}={Ď}_{\text{Actual}}*{\text{AKS}}_{\text{Nominal}}/{\text{AKS}}_{\text{Actual}}$$

The nominal dose rate at the prescription depth (Ď_Rx_) is related to the PDD as shown below.

$${Ď}_{\text{Rx}}={Ď}_{\text{Nominal}}*\text{PDD}$$

The nominal dwell time (t_Nominal_) is then computed from Ď_Rx _and the prescribed dose (D_prescribed_) as shown below.

$${\text{t}}_{\text{Nominal}}={\text{D}}_{\text{prescribed}}/{Ď}_{\text{Rx}}$$

The actual treatment time (t_actual_) is calculated prior to each treatment with measurement of the AKS_Actual _using real time temperature-pressure correction as shown below.

$${\text{t}}_{\text{actual}}={\text{t}}_{\text{nominal}}\;\left({\text{AKS}}_{\text{Nominal}}/{\text{AKS}}_{\text{Actual}}\right)$$

When customized shielding is used to optimize the dose to the PTV, a layer of high-density material, such as 1 mm lead or any commercially approved shielding can be used. The cut-out correction factors can be measured in the phantom as shown below.

$$\begin{array}{c} {\text{OF}}_{\text{CutoutCone A}}=\\ {\text{M}}_{\text{ConeA}(\text{uncollimated})}/{\text{M}}_{\text{ConeA }(\text{collimated})} \end{array}$$

The Nominal Dose Rate with the cutout should be adjusted by the Cone and Cut-out corrections, as shown below, so that the t_Nominal _can be calculated.

$$\begin{array}{c} {Ď}_{\text{Nominal},\text{ cutout}}=\\ {Ď}_{\text{Nominal},\text{ reference}}*{\text{OF}}_{\text{CutoutCone A}}*{\text{OF}}_{\text{ConeA}} \end{array}$$

### Treatment Delivery

The EBT system includes a miniature, electronic, high dose rate, low energy X-ray tube integrated into a flexible, multi-lumen catheter. This source produces X-rays of 50 keV maximum energy at the tip of the catheter. The EBT system also includes a mobile controller that contains the user interface and provides power to the X-ray source. Additional details on the EBT system are provided by Mehta, et al.[22] The EBT system with surface applicators was utilized to deliver a dose of 40.0 Gy in 8 fractions, 5 Gy per fraction. The treatments were delivered twice weekly with a minimum of a 48-hour interval between fractions. The prescription dose depth ranged from 3-7 mm based on the lesion depth. The PTV consisted of the lesion plus an acceptable margin. The margins ranged from 2 to 5 mm depending on treatment location. Initially, the 35 mm surface applicator was available prior to the other sizes, and commercially available cutout shielding was used under the surface applicator. Once all four applicator sizes were available, all four sizes were used.

All patients were treated outside of a linear accelerator vault in the CT simulator room. A flexible shield was placed over the applicator to minimize radiation exposure. Our site's standard of care was to provide a petrolatum ointment such as Eucerin^® ^Aquaphor^® ^ointment (Beiersdorf, Inc, Wilton, CT) to be applied to the treatment area three to four times per day during the duration of the radiation therapy treatments. Once the treatments were completed, patients were advised to apply an aloe vera gel to the treatment area through 1-month of follow up.

### Endpoints

Endpoints included treatment feasibility, acute safety outcomes, cosmetic results, and short-term efficacy. Treatment feasibility was defined as the successful delivery of the prescribed dose following the intended treatment schedule. Adverse events were collected during treatment and follow-up visits. Adverse events were categorized and graded according to the Common Terminology Criteria for Adverse Events (CTCAE) version 3 manual[23]. Efficacy was based on the rate of local recurrence. Cosmesis was rated as excellent, good, fair or poor using a standardized cosmesis scale[24]. Excellent was defined as no changes to slight atrophy or pigment change or slight hair loss or no changes to slight induration or loss of subcutaneous fat. Good was defined as patch atrophy, moderate telangiectasia, total hair loss; moderate fibrosis but asymptomatic, slight field contracture with less than 10% linear reduction. Fair was defined as marked atrophy, gross telangiectasia; severe induration or loss of subcutaneous tissue; field contracture greater than 10% linear measurement. Poor was defined as ulceration or necrosis[24].

## Results

### Patient Demographics

Thirty-seven patients with 44 cutaneous malignancies were treated with a HDR electronic brachytherapy system between July 2009 and March 2010. Table 1 represents the patient demographics for this study. Twenty-five (56.8%) lesions were BCC, 17 (38.6%) were SCC, one (2.3%) was Merkle Cell, and one (2.3%) was cutaneous T-cell lymphoma. The mean age of the patients was 72.5 years and ranged from 49 to 89 years. Thirty-nine of the 44 lesions (89%) were T1, one lesion (2.3%) was Tis, one lesion (2.3%) was T2, and three lesions were recurrences (6.8%) after prior surgical resection. Ninety-five percent of patients were Caucasian non-Hispanic, and 5% were Hispanic. Seventy-three percent of the patients were male.

**Table 1:** Demographics at Baseline

	Total	
**Histology**	**N**	**Percent**
Basal Cell	25	56.8%
Squamous cell	17	38.6%
Merckle Cell	1	2.3%
T-Cell Lymphoma	1	2.3%

**Tumor Stage**	**N**	**Percent**
Tis	1	2.3%
T1	39	88.6%
T2	1	2.3%
Recurrence	3	6.8%

**Ethnicity**	**N**	**Percent**
Caucasian/Non-Hispanic	35	94.6%
Hispanic	2	5.4%

**Gender**	**N**	**Percent**
Male	27	73.0%
Female	10	27.0%

**Lesion Locations**	**N**	**%**
Scalp	5	11.4%
Face	14	31.8%
Nose	16	36.4%
Extremity	4	9.1%
Ear	5	11.4%

All patients and all lesions underwent successful completion of treatment with the prescribed dose according to the treatment plan. All 44 lesions were treated with 40.0 Gy in eight fractions of 5.0 Gy each. Of the 44 lesions treated, 16 (36.4%) lesions were located on the nose, including the nasal ala, the nasal tip, and nostril. Five lesions (11%) were on the ear, which consisted of the pinna, anthelix, and ear lobe. Five lesions (11%) were located on the scalp, which included the top of the head and the post-auricular area. Fourteen lesions (32%) were on the face and included lesions on the forehead, cheek, temple, pre-auricular area, nasolabial fold. Four lesions (9%) were located on an extremity (Table 1).

The applicator sizes included 10 mm, used to treat 35% of the lesions, 20 mm, used to treat 25%, 35 mm, used to treat 43% of the lesions, and 50 mm, used for one patient (2%). The lesion sizes ranged from <1 cm to 5 cm as summarized in Table 2. Commercially available cutout shielding was used under the surface applicator to prevent delivery of the radiation therapy treatments to the skin beyond the PTV of 13 (29%) lesions. Six of 13 lesions were less than 1 cm in diameter, and 7 of the lesions were 1-2 cm in diameter. The prescription depth varied with the lesion depth and was 5 mm beyond the skin surface in 34 of the lesions and 3 mm depth in 9 of the lesions. One patient with a cutaneous T-cell lymphoma plaque had a deep lesion based on CT imaging which necessitated the prescription dose depth to be 7 mm for the first four fractions and 5 mm for the final four fractions as the tumor size began to decrease. The patients who underwent treatment with a prescription dose depth of 3 mm had lesions on the face in 6, the nose in 1, the ear in 1, and the scalp in 1. The mean treatment time was 6.8 minutes with a range from 4.7 to 13.8 minutes. The treatment times by applicator size and prescription dose depth are listed in Table 3.

**Table 2:** Applicator Sizes and Corresponding Lesion Size Range

Applicator Size	Lesion Size Range	Number of Lesions	Percent Of Total Lesions
10 mm	< 1 cm	13	29.5%

20 mm	1 cm	2	4.5%
	> 1 cm and ≤ 2 cm	9	20.5%

35 mm^1^	≤ 1 cm	6	13.6%
	> 1 cm and ≤ 2 cm	12	27.3%
	> 2 cm and ≤ 3 cm	1	2.3%

50 mm	5 cm	1	2.3%

mm = millimetre; cm = centimetre

^1^Cut-out shielding was used with the 35 mm applicator to treat 6 lesions ≤ 1 cm and 7 lesions > 1 cm and ≤ 2 cm.

**Table 3:** Treatment Times in Minutes By Applicator Size and Prescription Dose Depth

	10 mm Applicator		20 mm Applicator		35 mm Applicator	50 mm Applicator
**Prescription Dose Depth**	**3 mm**	**5 mm**	**3 mm**	**5 mm**	**5 mm**	**7 mm**

No. of Lesions	6	7	3	8	19	1
Treatment Time						
Mean	4.8	6.5	5.9	7.7	6.8	13.8
Min	4.7	5.3	5.6	7.0	5.8	13.8
Max	5.2	6.8	6.1	7.9	7.7	13.8

mm = millimetre; min = minimum; max = maximum

**Figure 4:**
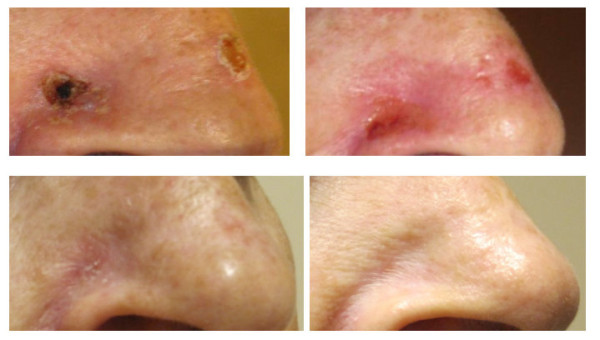
**EBT Treatment of Basal Cell Carcinoma**. Photo at pretreatment (top left), prior to fraction 7 of 8 (top right), at one-month follow up (bottom left), and at six months of follow up (bottom right).

Patients were followed for a median of 4.1 months (range 1-9 months). There have been no recurrences to date. Cosmetic outcomes were assessed as excellent, good, fair or poor according to Cox, et al., at each follow-up visit[24]. All patients had an excellent or good cosmetic outcome at each follow-up visit. At 1-month of follow up, 90% of patients had excellent cosmesis, and 10% had good cosmesis. At 3-months of follow up, 95% of the 19 evaluable patients had excellent cosmesis, and 5% had good cosmesis. An example of BCC treatment resulting in an excellent cosmetic outcome at 6 months post-radiation therapy is shown in Figure 4.

### Adverse Events

**Figure 5:**
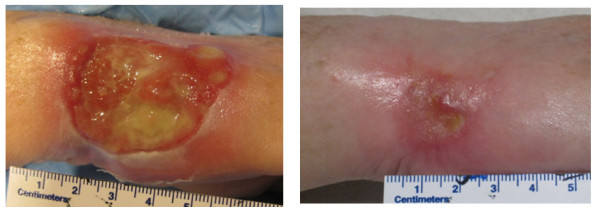
**EBT Treatment of Cutaneous T-cell Lymphoma**. Photo at pretreatment (left) and at two months of follow up (right).

All adverse events that occurred were CTCAE grade 1 or grade 2 regardless of prescription dose depth, which varied with lesion depth[23]. The prescription dose was 40 Gy for all patients. The patients who experienced grade 2 adverse events are listed in Table 4. For the patients who were treated with 40 Gy prescribed to a depth of 3 mm, all adverse events were grade 1. Seven of 8 (86%) adverse events are resolved, and one adverse event, erythema grade 1, was ongoing at 1-month of follow up and will undergo additional follow up. For the patients who underwent treatment of 40 Gy prescribed to a depth of 5 mm, 12 patients experienced grade 2 rash-dermatitis associated with radiation. All events have resolved except one, which improved to grade 1 at the 3-month follow-up visit and was ongoing at 6 months of follow up. One patient was treated for cutaneous T-cell lymphoma at a prescription depth of 7 mm. This patient experienced rash-dermatitis associated with radiation reported at fraction 7, and the adverse event was resolved at the 2-month follow-up visit. (Figure 5)

**Table 4:** Adverse Events with CTC AE Grade 2 Rash Dermatitis Associated With Radiation^1,^^2^

Subject	Onset	Improved to Grade 1	Resolved
1	Fraction 8	1 month	3 month
2	Fraction 5	Fraction 8	1 month
3	Fraction 4	1 month	3 month
4	Fraction 7	--------------	1 month
5	Fraction 4	1 month	6 month
6	1 month	3 month	Ongoing at 6 months
7, 8, 9, 10	Fraction 8	----------------	1 month
11, 12	Fraction 8	----------------	4 month

^1 ^All cases of rash dermatitis associated with radiation were assessed as CTCAE Grade 2, and all prescriptions were to a dose depth of 5 mm

^2 ^One case of rash dermatitis grade 2 occurred in the patient with cutaneous T-cell lymphoma. Prescription depth was 7 mm. The rash dermatitis improved to grade 1 at the 2-month follow-up visit.

## Discussion

The incidence of skin cancer is rapidly rising, and the treatment approach must be individualized based on specific risk factors and patient characteristics in order to achieve the most acceptable cosmetic and functional outcome. For those patients where surgical resection is not an ideal option or for those patients not interested in surgery, radiation therapy is a viable option. However, the traditional dose fractionation schemes lasting 5-7 weeks of daily radiation could result in this modality as a less desirable option for skin cancer patients. HDR brachytherapy offers a convenient treatment schedule for patients and is associated with excellent outcomes[15,16].

This report represents the initial experience using an electronic source for HDR brachytherapy with surface applicators for the treatment of NMSC. All patients received a hypofractionated course of EBT comparable to published treatment schedules for traditional HDR brachytherapy with a radioisotope source. The early results with EBT show similar outcomes to that with traditional HDR brachytherapy[15,16]. There were no patients with severe (Grade 3 or higher) toxicities. Additionally, all patients had a decline in or resolution of skin toxicities after 1 month of follow up. There have been no recurrences as of this publication with a mean follow up of 4.1 months (range 1-9 months).

Long-term control rates for NMSC treated with external beam radiation therapy, including superficial x-rays (45-100 kV), orthovoltage x-rays (100-250 kV), megavoltage photons, and electron beam radiation, range from 87% to 100% after a follow up of 2 to 5 years[9-14]. High dose rate brachytherapy with Ir-192 for NMSC has shown control rates of 92% to 98% after 5 to 10 years of follow up[15,16]. Other nonsurgical interventions for BCC and SCC include photodynamic therapy, laser therapy and a combination of the two. Photodynamic therapy for superficial BCC has a tumor-free rate of 91.2% to 94.8%, which increases to 99.0% when combined with erbium:yttrium aluminium garnet (Er:YAG) laser after a follow up of 3 months to 1 year[25,26]. Neodymium (Nd) and Nd:YAG lasers have been used in patients with facial NMSC; recurrence rates were 1.8% and 2.5% in BCC treated with pulsed Nd or Nd:YAG laser therapy and 4.4% in SCC treated with pulsed Nd laser after a follow up of 3 months to 5 years[27].

The dosimetric results for electronic brachytherapy and Ir-192 brachytherapy using surface applicators revealed similar depth dose profiles,[17-19] which could possibly explain the similar outcomes thus far. Additional follow-up data on EBT with surface applicators is needed in order to compare EBT with the long-term efficacy data of Ir-192 HDR brachytherapy with surface applicators. EBT with surface applicators does have a distinct beam flatness profile where nearly 100% of the dose encompasses the entire diameter of the surface applicator (Figure 3). This dosimetric advantage could potentially lead to reduced margin requirements for the treatment of cutaneous malignancies due to lack of penumbra[17-20]. Typically, at our institution, a 5 mm margin is utilized for these patients undergoing EBT using surface applicators. However, there are certain locations such as nasal tip, nasal ala, and facial areas near the eye, where a 5 mm margin is not feasible or desirable. Therefore, a reduced margin was utilized to account for these critical anatomic locations. A reduced treatment margin also could result in minimal toxicities with small treatment volumes compared to treating larger volumes as may be needed for other radiation modalities. These properties of EBT with surface applicators could lead to this modality becoming an acceptable treatment option for patients with NMSC.

## Conclusions

The early outcomes of electronic brachytherapy for the treatment of NMSC show acceptable acute toxicity and favorable early cosmesis. The hypofractionated approach provides patient convenience with effective early outcomes. Long-term follow up is in progress to further assess efficacy and cosmesis.

## Abbreviations

AE: Adverse Event; BCC: Basal Cell Carcinoma; cm: centimetre; CT: Computerized Tomography; CTC- Common Terminology Criteria; EBT: Electronic Brachytherapy; Er: erbium; GTV: Gross Tumor Volume; Gy: Gray; HDR: High Dose Rate; Ir: Iridium; kV: kilovoltage; Max: Maximum; Min: Minimum; mm: Millimetre; Nd: neodymium; NMSC: Non-melanoma Skin Cancer; PTV: Planning Target Volume; SCC: Squamous Cell Carcinoma; SD: Standard Deviation; TG 61: Task Group 61; Tis: Tumor in situ; T1: Tumor ≤ 2 cm in greatest dimension; T2: Tumor > 2 cm but not > 5 cm in greatest dimension; v3: Version 3; YAG: yttrium aluminium garnet

## Competing interests

AB was compensated by Xoft, Inc., for his role as Principal Investigator of this Retrospective Single Center Study. AB has received an honorarium payment for speaking at a radiation oncology conference on his experience using EBT for the treatment of NMSC. Xoft, Inc., paid the article processing charge.

## Authors' contributions

AB is the principal investigator of this retrospective study and was responsible for the development of the protocol and case report form; recording of the clinical data from the patient records; analysis of the data; and writing, final review, and approval of this manuscript. AL was responsible for the recording of treatment planning and treatment data and for the writing and final approval of this manuscript.

## Additional Information

This study will be presented at the 2010 Annual Meeting of the American Society for Therapeutic Radiology and Oncology in San Diego, CA.
